# In-Depth Profiling of Calcite Precipitation by Environmental Bacteria Reveals Fundamental Mechanistic Differences with Relevance to Application

**DOI:** 10.1128/AEM.02739-19

**Published:** 2020-03-18

**Authors:** Bianca J. Reeksting, Timothy D. Hoffmann, Linzhen Tan, Kevin Paine, Susanne Gebhard

**Affiliations:** a Department of Biology and Biochemistry, Milner Centre for Evolution, University of Bath, Bath, United Kingdom; b Department of Architecture and Civil Engineering, BRE Centre for Innovative Construction Materials, University of Bath, Bath, United Kingdom; North Carolina State University

**Keywords:** ureolysis, self-healing concrete, microbially induced calcite precipitation, *Bacillus*

## Abstract

Microbially induced calcite precipitation (MICP) has not only helped to shape our planet’s geological features but is also a promising technology to address environmental concerns in civil engineering applications. However, limited understanding of the biomineralization capacity of environmental bacteria impedes application. We therefore surveyed the environment for different mechanisms of precipitation across bacteria. The most fundamental difference was in ureolytic ability, where urease-positive bacteria caused rapid, widespread increases in pH, whereas nonureolytic strains produced such changes slowly and locally. These pH shifts correlated well with patterns of precipitation on solid medium. Strikingly, while both mechanisms led to high levels of precipitation, we observed clear differences in the precipitate. Ureolytic bacteria produced homogenous, inorganic fine crystals, whereas the crystals of nonureolytic strains were larger and had a mixed organic/inorganic composition. When representative strains were tested in application for crack healing in cement mortars, nonureolytic bacteria gave robust results, while ureolytic strains showed more variation. This may be explained by our observation that urease activity differed between growth conditions or by the different natures and therefore different material performances of the precipitates. Our results shed light on the breadth of biomineralization activity among environmental bacteria, an important step toward the rational design of bacterially based engineering solutions.

**IMPORTANCE** Biomineralization triggered by bacteria is important in the natural environment and has many applications in industry and in civil and geotechnical engineering. The diversity in biomineralization capabilities of environmental bacteria is, however, not well understood. This study surveyed environmental bacteria for their ability to precipitate calcium carbonate minerals and investigated both the mechanisms and the resulting crystals. We show that while urease activity leads to the fastest precipitation, it is by no means essential. Importantly, the same quantities of calcium carbonate are produced by nonureolytic bacteria, and the resulting crystals appear to have larger volumes and more organic components, which are likely beneficial in specific applications. Testing both precipitation mechanisms in a self-healing concrete application showed that nonureolytic bacteria delivered more robust results. Here, we performed a systematic study of the fundamental differences in biomineralization between environmental bacteria, and we provide important information for the design of bacterially based engineering solutions.

## Abstract (precis)

Biomineralization triggered by bacteria is important in the natural environment and has many applications in industry and in civil and geotechnical engineering. The diversity in biomineralization capabilities of environmental bacteria is, however, not well understood. This study surveyed environmental bacteria for their ability to precipitate calcium carbonate minerals and investigated both the mechanisms and the resulting crystals. We show that while urease activity leads to the fastest precipitation, it is by no means essential. Importantly, the same quantities of calcium carbonate are produced by nonureolytic bacteria, and the resulting crystals appear to have larger volumes and more organic components, which are likely beneficial in specific applications. Testing both precipitation mechanisms in a self-healing concrete application showed that nonureolytic bacteria delivered more robust results. Here, we performed a systematic study of the fundamental differences in biomineralization between environmental bacteria, and we provide important information for the design of bacterially based engineering solutions.

## INTRODUCTION

Environmental protests worldwide have highlighted a growing demand for action on the threat of climate change. Human activity has greatly contributed to global warming, currently causing an estimated 0.2°C increase per decade due to past and ongoing emissions (1). There is a clear need to reduce our carbon dioxide (CO_2_) emissions, as well as to find novel ways in which we can consume CO_2_ as a means of decreasing overall levels. Geological sequestration of CO_2_ from large point source emitters into carbonate minerals, such as dolomite and limestone, is an emerging technology that could mitigate increasing CO_2_ concentrations (2). Microorganisms can act as biomediators to enhance carbon capture (2) by microbially induced calcite precipitation (MICP). This process leads to precipitation of CO_2_ as calcium carbonate (CaCO_3_) as a result of microbial metabolism and has been occurring on a geological scale for more than 2.5 billion years (3). The formation by microbes of mineral carbonate structures, termed microbialites, is mediated by a large diversity of microorganisms, often working together in a consortium (4). Indeed, cyanobacteria, in association with heterotrophic bacteria, are thought to be the principal contributors to the production of carbonate rocks during almost 70% of Earth’s history (3.5 to 0.5 billion years) (3).

The success of MICP in biological engineering of the environment has led to the investigation of its potential for exploitation in other areas. These areas include bioremediation of heavy metal-contaminated soils via immobilization of metals in precipitates (5, 6) and wastewater treatment using this technology to remove excess calcium. Geotechnical engineering represents another area of interest, in which MICP can improve soil properties by precipitating CaCO_3_ to bind sand particles together. Utilization of MICP in the construction industry is another rapidly emerging field. The production and maintenance of concrete bear heavy environmental and economic costs, with cement production contributing up to 5 to 8% of global anthropogenic CO_2_ emissions (7, 8). Moreover, reinforced concrete structures face durability issues caused by cracking and subsequent ingress of water and ions such as chlorides. This leads to corrosion of internal steel reinforcement and eventual structural failure. MICP can seal such cracks in concrete and reduce permeability to damaging substances, thereby extending the life span of structures and reducing the environmental burden caused by construction of replacement structures. This broad range of applications necessitates an understanding of how bacteria precipitate calcite and what the factors are that may limit their performance.

Most bacteria are capable of MICP under suitable conditions (9), with key factors such as calcium concentration, concentration of dissolved inorganic carbon (DIC), pH, and availability of crystal nucleation sites affecting precipitation (10). Bacterial metabolism results in an increase in both pH and DIC (through aerobic or anaerobic oxidation of organic compounds) and in changes in solution chemistry (11). This can lead to the oversaturation of Ca^2+^ and CO_3_^2−^ ions to facilitate the formation of calcium carbonate precipitates (11). The negatively charged cell surfaces of bacteria further promote precipitation by attracting calcium ions and acting as nucleation sites for crystal formation (12).

To date, the majority of studies of MICP have focused on ureolytic bacteria (12). These are associated with high rates of CaCO_3_ precipitation (2), caused by hydrolysis of urea [CO(NH_2_)_2_] to ammonia (NH_3_) and carbonic acid (H_2_CO_3_) (2). The reaction of ammonia with water to ammonium (NH_4_^+^) and hydroxide ions (OH^−^) causes an upshift in pH, while carbonic acid dissociates to generate bicarbonate (HCO_3_^−^). In the presence of calcium, this leads to the formation of a calcium carbonate (CaCO_3_) precipitate (equations 1 and 2).

(1) $$\text{CO}{\left({\text{NH}}_{\text{2}}\right)}_{\text{2}}+{\text{2H}}_{\text{2}}\text{O}\to {\text{2NH}}_{\text{4}}{}^{+}+{\text{CO}}_{\text{3}}{}^{\text{2}-}$$

(2) $${\text{Ca}}^{\text{2}+}+{\text{CO}}_{\text{3}}{}^{\text{2}-}\to {\text{CaCO}}_{\text{3}}\left(\text{s}\right)$$

Ureolytic MICP has been used in a range of applications. Positive outcomes were obtained in the improvement of soil properties; however, nonuniform rates of precipitation throughout the samples led to varying results (13). In addition, the requirement for injection of the healing agent into the ground created problems in which the rapid precipitation initiated by urease activity caused clogging at the injection site (14). In other applications, such as crack sealing in cementitious materials, ureolytic MICP has been shown to promote healing (15, 16). However, because of the dependency of the reaction on enzymatic activity, factors such as low temperature markedly decreased the efficiency of the reaction (17). In addition, ureolytic MICP requires the presence of urea, which may not always be feasible to provide, and the release of ammonia could contribute to environmental nitrogen loading (18).

To work around the challenges associated with ureolytic bacteria, the use of other metabolic pathways needs consideration. The high efficiency of ureolytic MICP has resulted in the assumption that nonureolytic pathways cannot yield similarly high levels of precipitation and are thus an inferior strategy to achieve precipitation. This has limited our understanding of alternative pathways, and the factors affecting efficient calcite precipitation in nonureolytic bacteria are not clear. Studies on self-healing concrete using alkaliphilic nonureolytic bacteria such as Bacillus pseudofirmus and Bacillus cohnii have shown great promise and have led to similar levels of self-healing at the laboratory scale as ureolytic bacteria (19, 20). To fully assess the potential of MICP for industrial and environmental use, detailed understanding of the microorganism, mechanism of precipitation, and application is therefore important.

Most studies of MICP have focused on a small number of species and have thus restricted our understanding of the mechanisms of precipitation across bacteria. In addition, many previous studies have been primarily application driven and have not always explored the deeper workings of how the bacteria precipitate minerals and what influences this ability. To build a strong basis on which to develop our understanding of MICP, we took a broad view here and surveyed in detail the ability of environmental bacteria to precipitate calcite. Focusing on bacteria that could grow in the alkaline conditions typical of high-calcium environments, we assessed the prevalence of MICP capability in environmental bacteria, their mechanisms of precipitation and resulting crystal morphologies, and their suitability for application in self-healing concrete. Our study provides an understanding of the environmental potential for MICP, which is not only relevant to geomicrobiology but will facilitate a more design-based approach to industrial application of MICP.

## RESULTS AND DISCUSSION

### Calcite precipitation ability of environmental isolates.

To gain a broader understanding of the capability of bacteria to precipitate calcium carbonate, we first performed a survey of a range of environments. In this, we focused on sites expected to be calcium rich, such as exposed limestone, caves, and soils in areas with a limestone bedrock, within the southwestern United Kingdom. Our isolation strategy targeted spore-forming bacteria, as their stress tolerance makes them more universally useful in potential applications. Moreover, as many of the high-calcium environments are in the alkaline pH range, we specifically sought isolates that tolerate such conditions. Interestingly, the majority (89%) of colonies obtained during primary isolations had visible crystal formation on the surface that was indicative of biomineralization. This supports previous observations that this is a common trait among bacteria (9). Of 74 isolates able to grow at pH 9, 31 displayed some growth at pH 10 on 0.25× B4 minimal medium and were chosen for further characterization.

**FIG 1:**
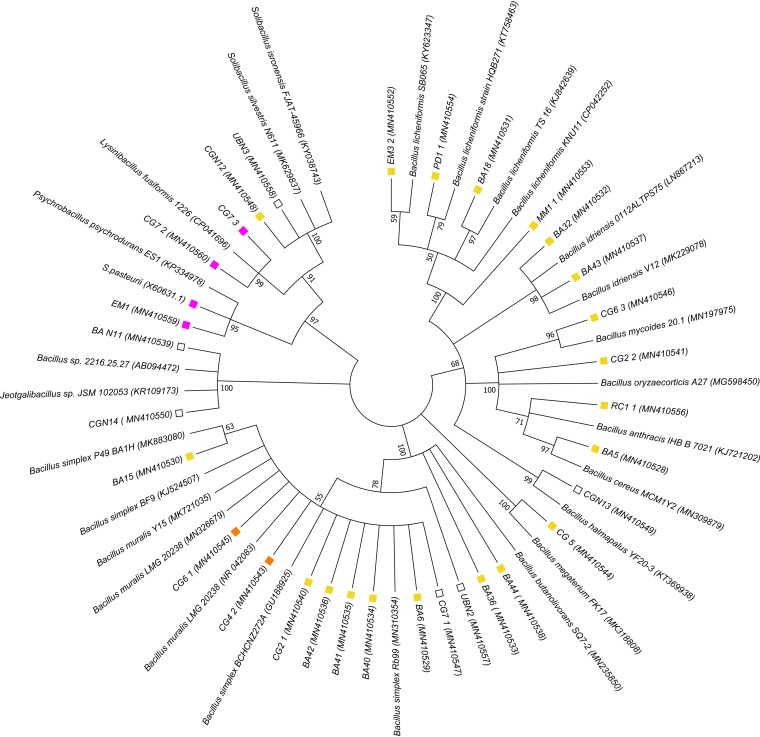
Phylogenetic analysis of selected calcite-precipitating isolates and their closest relatives. Yellow boxes, nonureolytic isolates; orange boxes, isolates with inducible ureolytic activity; pink boxes, isolates that utilize urea as a primary nitrogen source; unfilled boxes, unknown ureolytic ability. The ureolytic potential of closest relatives is not shown. GenBank accession numbers are indicated in parentheses. The percentages of trees in which associated taxa clustered together are shown next to the branches (1,000 bootstraps).

Preliminary identification based on partial sequencing of the 16S rRNA genes assigned all of the isolates to the order Bacillales (Fig. 1). The selection of spore formers by heat treatment can account for the prevalence of *Bacillales* spp., with most isolates grouping within the Bacillus genus. The predominant species represented were Bacillus licheniformis and Bacillus muralis. Sporosarcina pasteurii was included in the analyses since it represents the paradigm organism for industrial application of calcite precipitation. Interestingly, five of our isolates grouped within the same clade as S. pasteurii (Fig. 1).

### Mechanistic differences in calcite precipitation between isolates.

**FIG 2:**
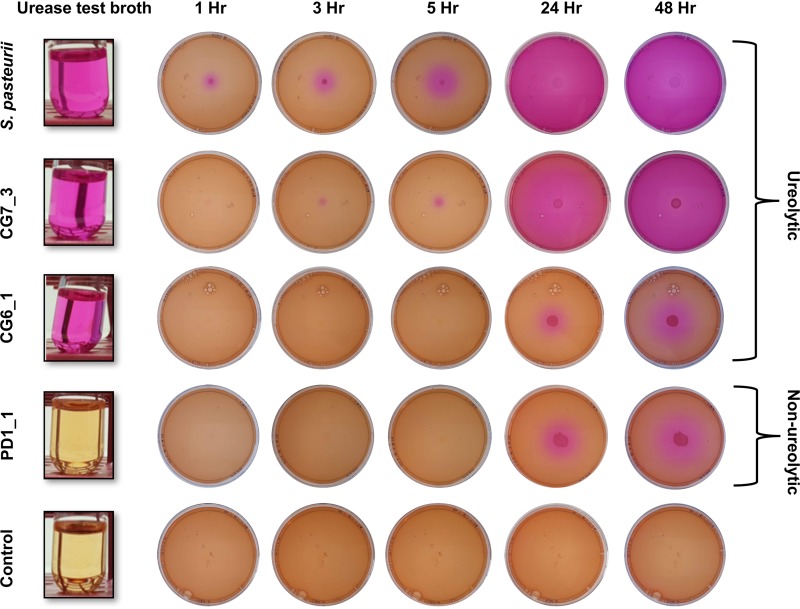
Diversity in ureolytic activity across environmental isolates. (Left) Urease test broth inoculated with the strains indicated to the left. (Right) Nutrient agar plates supplemented with phenol red, calcium acetate, and urea were inoculated in the center with suspensions of the same strains. “Control” images show uninoculated media. Pink shading indicates the pH change caused by ammonia release from ureolytic activity.

The isolation strategy, with the exception of looking for alkali-tolerant spore formers, was unbiased for particular metabolic properties of the bacteria. A key feature of many known calcite precipitators is ureolysis, which leads to a rapid pH change in the environment due to the release of ammonia and subsequent production of OH^−^. We therefore tested each of our isolates for its ability to cleave urea, indicated by a pink color change in test broth containing urea and phenol red (Fig. 2 and Fig. S2). Five out of the 31 isolates taken forward for further characterization were ureolytic. Two of these ureolytic isolates (CG4_2 and CG6_1) were most closely related to B. muralis, while two others (CG7_2 and CG7_3) were identified as Lysinibacillus fusiformis. The final isolate (EM1) was identified as Psychrobacillus psychrodurans and was most closely related to *S. pasteurii*. With the exceptions of CG4_2 and CG6_1, the other ureolytic isolates formed a monophyletic clade with the strongly ureolytic species *S. pasteurii* (Fig. 1).

To determine the effects of ureolysis and the associated pH change on crystal formation, we repeated the experiment on solid medium. Nonureolytic strains showed only small changes in pH, as indicated by local color changes in the immediate vicinity of the bacterial colony. This local shift in pH close to bacterial growth is most likely due to degradation of the amino acid components of the growth medium, leading to local release of ammonia (Fig. 2, isolate PD1_1; Fig. S2). In contrast, a rapid and widespread increase in pH was apparent in the model ureolytic bacterium *S. pasteurii*. Indeed, an increase in pH, as indicated by a color change from orange to pink, was visible as early as 1 h after inoculation of the plate with *S. pasteurii*, and the pH of the entire plate was alkaline by 24 h (Fig. 2). Similarly, the closely related ureolytic isolate CG7_3 caused a rapid increase in pH, although the rate of pH change was slightly slower than seen in *S. pasteurii* (Fig. 2). The same behavior was observed for isolate EM1 (Fig. S2).

**FIG 3:**
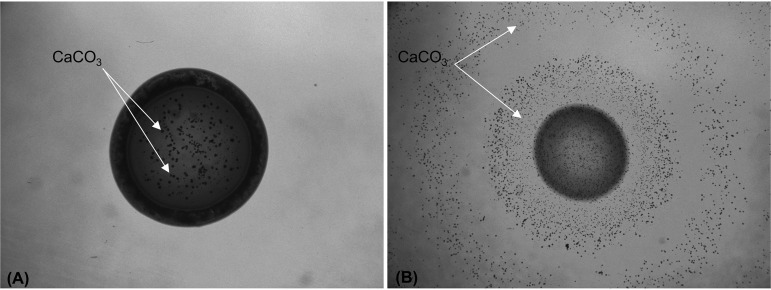
Crystal formation correlates with pH changes in nonureolytic and ureolytic bacteria. (A) Nonureolytic isolate PD1_1 shows localized crystals on the bacterial colony. (B) Ureolytic *S. pasteurii* demonstrates crystal precipitation throughout the medium, corresponding to pH changes that occur throughout the medium. Arrows indicate CaCO_3_ crystals.

We next investigated the patterns of calcium carbonate precipitation on the same agar plates and found that crystal formation correlated well with pH changes within the medium. In nonureolytic strains, crystals were visible only on the surface of the colony, corresponding to the observed localized pH change (Fig. 3A). In contrast, with ureolytic strains, crystal precipitation reflected the global pH change and occurred throughout the medium (Fig. 3B). The precipitation of calcium carbonate crystals throughout the medium was likely due to a change in the saturation kinetics in the medium, resulting from the rapid increase in pH caused by the urease-mediated release of ammonia from urea. Indeed, when urea was omitted for isolate CG7_3, only the localized pH changes associated with nonureolytic isolates were seen (Fig. S3). In addition, crystals were only visible on the surface of the colony when urea was omitted (Fig. S4), indicating that there are mechanistic differences in calcite precipitation under ureolytic and nonureolytic conditions.

**FIG 4:**
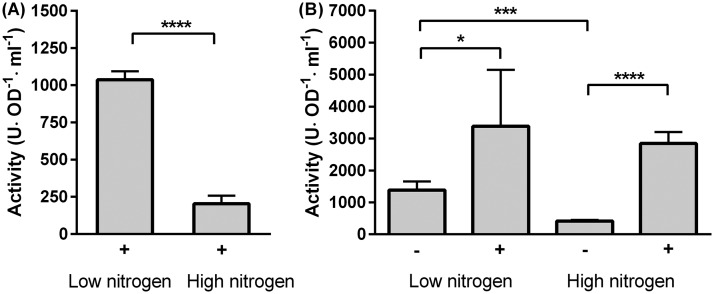
Regulation of urease activity by environmental nitrogen and urea. Isolates CG6_1 (A) and CG7_3 (B) cells were grown in high- or low-nitrogen conditions in the presence (+) or absence (−) of urea. Error bars represent standard deviation from 2 or 3 biological replicates with 1 or 2 technical replicates each. Significance was tested using an unpaired *t* test (A) and one-way analysis of variance (ANOVA) followed by unpaired *t* test (B). *, *P* < 0.05; ***, *P* < 0.001; ****, *P* < 0.0001.

Interestingly, two isolates that had tested positive for ureolysis in the test broth, CG6_1 and CG4_2, did not produce the characteristic rapid rise in pH and associated crystal precipitation when grown on solid medium (Fig. 2 and S2). A possible explanation for this discrepancy might be that in these isolates, urease activity is inducible in response to a cue that was present in test broth, but not in the solid medium. The main difference between the two media was the nitrogen content, which was very low in urease test broth (0.01% [wt/vol] yeast extract and 2% [wt/vol] urea), but high in the solid medium (0.2% [wt/vol] yeast extract, 0.5% [wt/vol] peptone, 1% [wt/vol] Lab-Lemco powder, and 2% [wt/vol] urea). Nitrogen metabolism and urease production are tightly controlled in bacteria (21). One mechanism of regulation of urease is by repression of activity in the presence of ammonia- or nitrogen-rich compounds and derepression of synthesis when nitrogen levels are low (21). We therefore tested to see whether nitrogen levels in the medium were having an effect on urease activity in these strains. Isolate CG6_1 was grown in the presence of urea in medium with either low (0.02% yeast extract, 0.0125% peptone, and 2% urea) or high (0.2% yeast extract, 0.125% peptone, and 2% urea) nitrogen content, and urease activity was quantified (Fig. 4A). Urease activity in CG6_1 was high during growth in low-nitrogen conditions, whereas significantly lower levels were detected in high-nitrogen conditions. This suggests that urease is active in CG6_1 only when the nitrogen supply is limited. This is clearly a type of behavior distinct from that seen in CG7_3 and *S. pasteurii*, which both utilize urea rapidly regardless of the nitrogen composition of the media (Fig. 2). However, while the overall urease activity of CG7_3 appeared similar to that of *S. pasteurii* on solid medium (Fig. 2), this isolate can grow well in the absence of urea (Fig. S3 and S4), while *S. pasteurii* cannot. This suggests that CG7_3 does not always require urease activity and hence may possess a means of controlling urease gene expression in a way that it is induced only in the presence of urea. We therefore tested the activity of whole cells grown in low- and high-nitrogen conditions, as before, but also compared cells grown in the presence and absence of urea (Fig. 4B). When CG7_3 was grown in the absence of urea, it displayed medium urease activity under low-nitrogen conditions, which was further repressed by high-nitrogen conditions. However, when cells were grown in the presence of urea, urease activity was high regardless of nitrogen conditions. Therefore, while urease activity in CG7_3 did show a partial response to nitrogen limitation, this was overcome by incubation with urea, which clearly acted as the main inducer and may be the preferred nitrogen source for this bacterium under the chosen growth conditions. Taken together, our results indicate that there is a continuum of ureolytic activity in environmental bacteria and that this, as seen above (Fig. 2 and 3), will have an impact on crystal formation and biomineralization by different isolates. It should be noted that we cannot rule out the possibility that isolates designated here as nonureolytic could harbor urease genes that were not expressed under our growth conditions and therefore would not have been detected with the phenotypic assays used.

### Effect of ureolysis on crystal formation.

**FIG 5:**
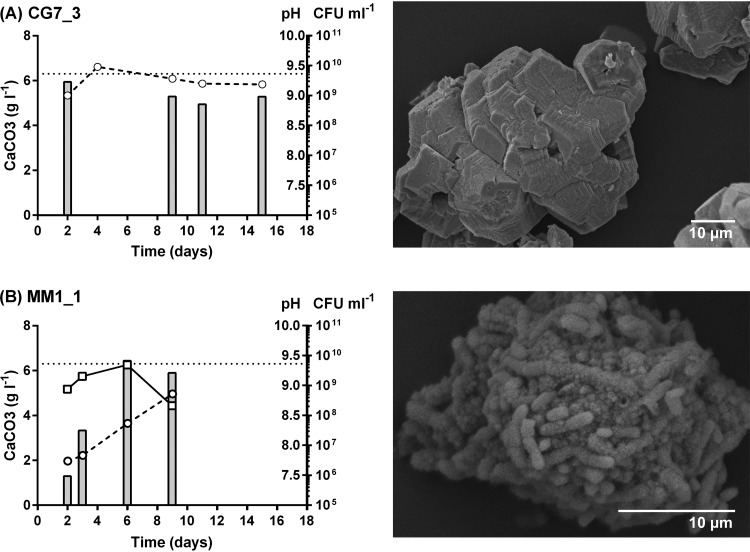
Calcite precipitation kinetics and crystal morphology in ureolytic CG7_3 and nonureolytic MM1_1. The ureolytic strain CG7_3 (A) and the nonureolytic strain MM1_1 (B) were grown in LB medium supplemented with urea (20 g · liter^−1^) and Ca(OAc)_2_ (10 g · liter^−1^), and precipitation of insoluble calcium carbonate (bars, g · liter^−1^), pH changes (circles), and changes in cell number (boxes, CFU · ml^−1^) were monitored over time (days). Maximum theoretical level of precipitation is indicated (dotted line). (Right) Electron micrographs of representative precipitate taken at day 9.

Having established that crystal formation on solid medium is very different between ureolytic and nonureolytic strains and that there are notable variations in ureolytic activity between environmental isolates, we wanted to understand how ureolysis influences crystal formation in more detail. To investigate this, we studied the kinetics of precipitation in our set of isolates. Growing isolates in liquid culture with calcium over several days allowed us to monitor the rates and total amounts of precipitate formed and correlate this to bacterial growth and global pH changes. Growth of ureolytic isolates caused rapid alkalinization of the medium, reaching pH 9 within 2 days, the first time point assayed. This resulted in complete precipitation of the available calcium in the medium on the same time scale, with no further change observed in 2 weeks of incubation (Fig. 5A and Fig. S5, *S. pasteurii*). Growth of nonureolytic bacteria caused a more gradual increase in pH, approaching a pH of 9 only in the second week of incubation (Fig. 5B and Fig. S5). Precipitation of calcium in the medium was also more gradual, although both ureolytic and nonureolytic isolates attained the maximum theoretical levels of precipitation by the end of the first week. Strikingly, we observed noticeable differences in cell numbers over the experimental period. Viable cell counts decreased dramatically in ureolytic isolates and were undetectable within 2 days (Fig. 5A), likely due to high ammonia concentrations, resulting from the cleavage of urea, in the medium of these isolates. Alternatively, these cells may have been encased by calcium carbonate, preventing their further growth and division. In contrast, for nonureolytic isolates, cell numbers gradually increased before declining again over the course of prolonged incubation (Fig. 5B). These differences in viability over time between ureolytic and nonureolytic bacteria may have implications for application and should especially be considered in cases where the continued presence of viable cells will be required for multiple cycles of precipitation.

Considering that the total amounts of precipitate were similar in ureolytic and nonureolytic isolates, we next investigated whether the differences in the speed of precipitation had any effects on the resulting crystals. As an initial observation, the precipitate recovered from ureolytic strains was a much finer powder than that recovered from nonureolytic strains. Energy-dispersive X-ray (EDX) analyses of the different precipitates confirmed that all crystals tested were composed of calcium carbonate (Fig. S6). However, further investigation using scanning electron microscopy (SEM) revealed that the crystal morphology differed between ureolytic and nonureolytic strains (Fig. 5). When precipitation was rapid, such as in ureolytic strain CG7_3 (Fig. 5) and *S. pasteurii* (Fig. S5 and S7), inorganic, homogenous crystals were produced. Nonureolytic strains such as MM1_1 that precipitated calcium carbonate more slowly produced crystals containing significant proportions of bacterial cells and appeared more “organic” in nature (Fig. 5B). Similarly, under these conditions, CG4_2 and CG6_1 displayed the gradual increases in pH (Fig. S5) and more organic precipitate characteristic of nonureolytic strains (Fig. S7). Considering that the assay conditions used for this experiment included high nitrogen, it can be expected that urease activity was repressed under these conditions and resulted in strains behaving like nonureolytic strains. This differing appearance of precipitates in ureolytic versus nonureolytic conditions was consistently observed across all isolates tested in this assay (Fig. S5 and S7). These results show that the differences in ureolytic activity across our environmental isolates not only affected the kinetics of biomineralization but also had a clear impact on the crystal morphologies in the resulting precipitate.

### Profound effects of urease activity on precipitation of calcium carbonate.

**FIG 6:**
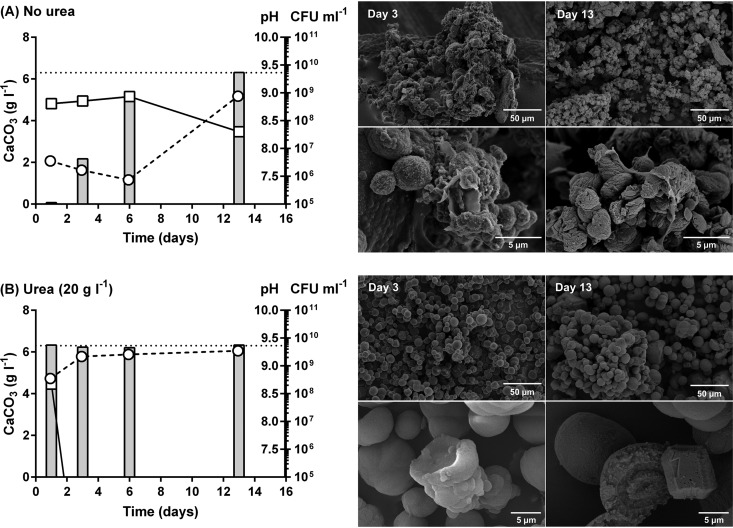
Comparison of ureolytic and nonureolytic mechanisms of calcite precipitation in CG7_3. The ureolytic strain CG7_3 was grown in LB medium supplemented with Ca(OAc)_2_ (10 g · liter^−1^) in the absence (A) and presence (B) of urea (20 g · liter^−1^). Precipitation of insoluble calcium carbonate (bars, g · liter^−1^), pH changes (circles), and changes in cell number (boxes, CFU · ml^−1^) were monitored over time (days). Maximum theoretical level of precipitation is indicated (dotted line). (Right) Electron micrographs of representative precipitate taken at days 3 and 13.

To exclude the possibility that the observed differences in calcium carbonate precipitation were simply due to strain-to-strain variation among our isolates, we exploited the fact that CG7_3 was capable of switching between ureolytic and nonureolytic states depending on the availability of urea (Fig. 2 and Fig. S3). This allowed us to directly investigate the impact of ureolysis on biomineralization in a single strain. When CG7_3 was grown in the absence of urea, there was a gradual increase in pH and calcium carbonate precipitation like that seen in nonureolytic strains (Fig. 6A). In contrast, in broths supplemented with urea, we observed a rapid increase in pH and associated calcium carbonate precipitation characteristic of the ureolytic strains studied above (Fig. 6B). Viable cell counts also dropped dramatically in CG7_3 utilizing urea (Fig. 6B), whereas in the absence of urea, CG7_3 cell numbers remained relatively stable over the first 10 days and declined by 13 days, likely due to prolonged nutrient depletion. SEM analysis of the precipitates over the time course of the study confirmed our previous observations (Fig. 5) that urease activity was correlated with the rapid precipitation of homogenous inorganic crystals (Fig. 6B). We noticed that, when assessed over time, the initial precipitate consisted of spherical calcium carbonate, typical of the polymorph vaterite, followed by the eventual conversion into the rhombohedral morphology associated with calcite, the more stable polymorph (Fig. 6B and Fig. S8 and S9). In comparison, in the absence of urea, CG7_3 precipitated calcium carbonate more gradually, and these precipitates were very organic in appearance (Fig. 6A and Fig. S8 and S9). Moreover, these “organic” crystals were often much larger than the inorganic spheres produced in the presence of urea, likely due to aggregation via interspersed bacterial cells. As these experiments were all performed on the same bacterial isolate, our observations are a direct reflection of the effects of urease activity on calcite precipitation. Although both pathways produced similar quantities of precipitate, there were major differences in precipitate morphologies, number of viable bacteria, and pH of the bulk phase. Our study thus reports a systematic investigation of the fundamental differences in biomineralization between different environmental bacteria. Both ureolytic and nonureolytic bacteria are currently being developed for a range of industrial applications (22). The findings reported here may therefore offer a key step toward a rational design approach to choosing which mechanism of biomineralization is better suited to specific industrial applications.

### Industrial relevance of different calcite precipitation strategies.

**FIG 7:**
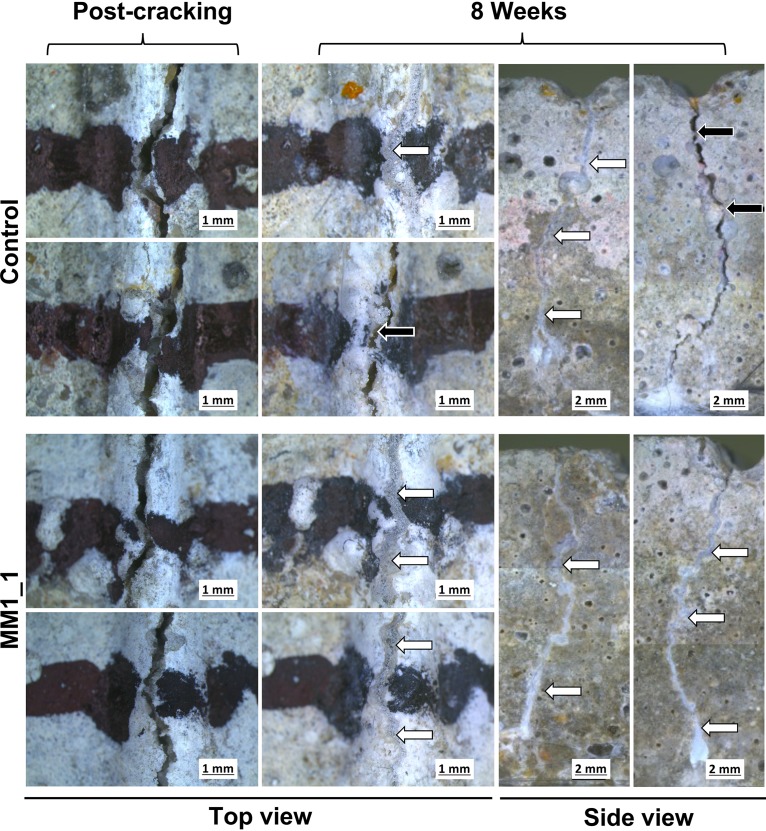
Crack closure in control mortars and in mortars containing nonureolytic bacteria. Cracks in mortar specimens are shown immediately postcracking and after 8 weeks of healing. Cracks were marked with black pen to allow the same region to be monitored over time. Two independent regions along the top of the mortar were monitored per sample. Side views show the cracks down both sides of the mortar at 8 weeks. White arrows, complete crack closure; black arrows, incomplete crack closure. Image manipulations were restricted to adjusting contrast and exposure. Due to the limited imaging area of the camera, side-view images were manually assembled from multiple photographs of overlapping sections of the specimen.

**FIG 8:**
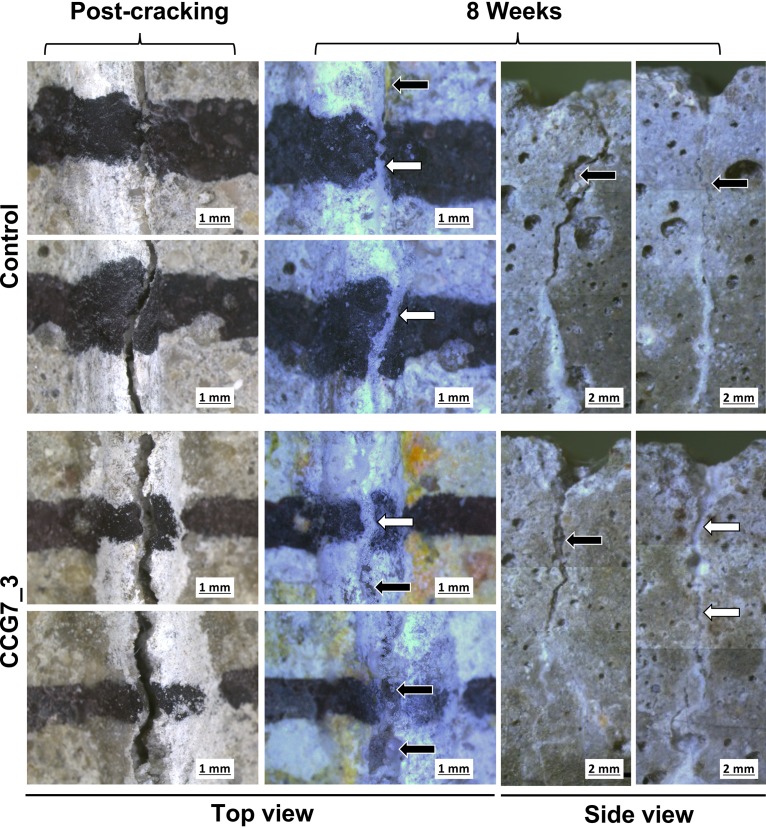
Crack closure in control mortars and in mortars containing ureolytic bacteria. Cracks in mortar specimens are shown immediately postcracking and after 8 weeks of healing. Cracks were marked with black pen to allow the same region to be monitored over time. Two independent regions along the top of the mortar were monitored per sample. Side views show the cracks down both sides of the mortar at 8 weeks. White arrows, complete crack closure; black arrows, incomplete crack closure. Image manipulations were restricted to adjusting contrast and exposure. Due to the limited imaging area of the camera, side-view images were manually assembled from multiple photographs of overlapping sections of the specimen.

To test if the differences in the biomineralization mechanism translate to an applied setting, we next assessed the performance of ureolytic and nonureolytic strains in self-healing concrete applications, using cement mortars as our test system. We produced spores for each strain, which were then encapsulated in lightweight aggregate before casting them in mortars together with yeast extract and calcium nitrate, as well as urea in the case of ureolytic strains, to provide nutrients for bacterial growth and sufficient calcium for biomineralization. Mortars were cured and then cracked under 3-point bending to obtain a target crack width of 500 μm. The self-healing process was subsequently monitored over 8 weeks. Autogenous healing, which occurs to some degree due to cement hydration, was seen in control mortars that lacked any bacterial spores (Fig. 7 and 8). This healing was mostly observed along the top edge of the crack and rarely extended down the sides of the mortar prism. Mortars containing either ureolytic or nonureolytic bacteria also displayed crack healing, and this generally extended across the top, as well as down the sides, of the crack (Fig. 7 and 8). However, healing in mortars containing ureolytic bacteria was less regular than seen with nonureolytic strains, with the sides of cracks not consistently sealing (Fig. 8).

**FIG 9:**
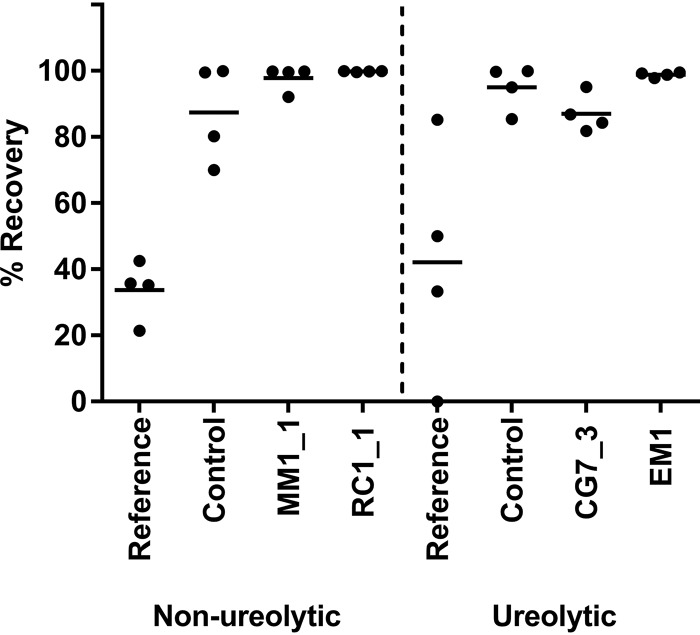
Recovery of watertightness in mortars after 2 months of bacterially based self-healing. Water flow through mortars containing ureolytic (CG7_3 and EM1) or nonureolytic (MM1_1 and RC1_1) bacteria was determined immediately after cracking and following 8 weeks of healing. Reference mortars contained no additives; control mortars contained yeast extract and calcium nitrate, but no bacteria. Recovery of watertightness was determined as a percentage relative to the freshly cracked specimen (0%, no change in water flow; 100%, complete inhibition of water flow). Data are shown for four specimens per condition from two separate cracking/healing experiments.

The key aim of bacterially induced self-healing of concrete is to reestablish watertightness of the structure to prevent water ingress and subsequent corrosion of steel reinforcement. While visual inspection allowed an initial assessment of the healing process, we next sought to test watertightness of our mortars using water flow tests following 8 weeks of healing. Recovery of watertightness in mortars containing only the standard cement mix with no nutrients or additional calcium showed variable recovery of watertightness, generally close to 40% (Fig. 9, “Reference”). Control mortars containing yeast extract and calcium nitrate, but without bacterial spores, showed a much higher recovery rate (averaging 87 to 95%). This recovery is likely because of a combination of autogenous healing and possible presence of environmental bacteria, which may be able to utilize the yeast extract and thus contribute to precipitation. As the mortars were not made or kept in a sterile fashion in order to more closely reflect industrial application, contamination with such environmental bacteria must be considered likely. The mortars containing nonureolytic bacteria showed a strikingly consistent recovery in watertightness that led to near-complete resistance to water flow, with all specimens healing to over 90% and many reaching close to 100% healing (Fig. 9). Interestingly, while mortars containing ureolytic bacteria also showed good restoration of watertightness, there was more variation in the degree of healing obtained. EM1 performed as well as the nonureolytic strains, but CG7_3 showed lower overall recovery values (mean, 87%), which were similar to those of the controls lacking encapsulated bacteria. This discrepancy in results may reflect the variability in ureolytic activity in these strains. Given the clear dependence on growth conditions in some strains described above, it is difficult to predict the degree of ureolytic ability displayed in cement mortars. An alternative explanation may be that the rapid precipitation and small crystal size observed in ureolytic isolates do not reliably lead to retention of the precipitate within the crack and thus may not perform as reliably in self-healing as the larger aggregates with organic components of the nonureolytic strains. It will be interesting to investigate the details of material performance following healing with both types of bacteria to fully understand the implications of the different mechanisms of precipitation.

### Concluding remarks.

The environment represents a large reservoir of potential in terms of exploiting bacteria for commercial use of MICP. There have been numerous studies on MICP for various applications; however, most of these have been restricted to the use of a single or small number of species and were very application specific. This limits our understanding of the precipitation capabilities of environmental bacteria more generally. In order to establish a stronger knowledge base to facilitate new and specialized technical applications, we surveyed the ability of environmental bacteria to precipitate calcite. We found that the majority of isolates were capable of biomineralization, in line with previous results (9). This is most likely due to their ability, through metabolic processes, to create a microenvironment around the cell that aids in the precipitation process. The electronegative nature of their cell surface facilitates crystal deposition on the surface (23), making precipitation more favorable. Our detailed characterization of biomineralization by our set of isolates, however, revealed fundamental differences in the way in which different bacteria precipitate calcium carbonate.

Ureolytic bacteria rapidly precipitate calcium carbonate, which has led to ureolysis becoming one of the main pathways used in MICP applications to date. However, in our study, only 16% of isolates were able to cleave urea, and even among these there was a marked diversity in ureolytic activity. Differences in how isolates regulate urease activity were seen, with some responding to low nitrogen levels, while others responded to urea as a primary inducer. This was in striking contrast to the constitutive high urease activity of *S. pasteurii*, one of the most frequently utilized MICP-capable bacteria to date (12, 24, 25). The performance of ureolytic bacteria will therefore depend on the precise conditions encountered in their environment, and this should be carefully considered when choosing the bacterial species for a given application. The importance of this point is further emphasized by our finding that ureolysis affected crystal formation both in terms of kinetics and in the morphology of the precipitate.

The majority of our isolates precipitated calcium carbonate without possessing ureolytic activity. In contrast to initial expectations that this mechanism may lead to the formation of small amounts of precipitate, we found that all of our isolates were capable of precipitating the total amount of calcium supplied in the growth media, although this process took more time with nonureolytic strains. Importantly, we showed that the precipitate formed by the nonureolytic bacteria consisted of larger, mixed organic/inorganic crystals. This was in good agreement with our initial observation that precipitation in these bacteria on solid medium occurred only on top of the colony and therefore likely involved the physical presence of cells. The mixed nature of the precipitate means that for the same amount of calcium being used, a larger volume of precipitate can be formed. This could present a major advantage in industrial application, where often only a limited amount of calcium is available to fill a relatively large space, such as in self-healing concrete.

When we tested the performance of our isolates in self-healing of cracked cement mortars, we found that, indeed, nonureolytic bacteria caused more consistent recovery of watertightness and more complete healing. The ureolytic bacteria tested showed less consistency in performance, although one strain gave similar results to those with the nonureolytic isolates. It is difficult to ascertain whether these inconsistencies in behavior are due to differences in ureolytic activity in the applied setting or are a consequence of the purely inorganic precipitate formed by ureolytic strains. It seems plausible that the supply of yeast extract as part of the cement mix will lead to a low-nitrogen environment conducive to urease gene expression, and the presence of urea should ensure activity in those strains that are urea responsive. While further experimentation is needed to determine the precise conditions encountered by the bacteria within hardened cement mortars, as well as the material properties of the precipitate formed, in applications where nutritional supply is hard to control, the use of nonureolytic bacteria may give more robust and reliable performance.

Self-healing concrete of course represents only one of many potential applications of MICP, and each application will have its own challenges. For example, in spray-on applications such as the restoration of existing structures and historic buildings, the rapid precipitation of large amounts of precipitate through ureolysis may be advantageous. In contrast, during soil stabilization, in which bacteria and their substrates are pumped into the ground, high rates of ureolysis could result in premature precipitation and lead to blockages at the injection site, as reported previously (14). In this case, it would be more beneficial to use microbes that are nonureolytic, or that switch to ureolysis at a later time point, as seen in our isolates that responded to low environmental nitrogen.

In summary, most bacteria have the ability to precipitate calcium carbonate given the right conditions, and the most suitable bacteria to use will be application dependent. While most previous studies have focused on one or two isolates, we show here the plethora of MICP activities and capabilities the environment has to offer as a “talent pool” that can offer bespoke solutions to many different applications. While we focused here on high-pH conditions often encountered in high-calcium environments, it can be assumed that an even wider reservoir of biomineralization can be discovered by testing other environments and different isolation strategies. MICP-based technologies may offer solutions to problems caused by a rising global population and may even be used to mitigate global warming. The increasing demand for infrastructure, as well as the need to engineer land on which to build it, results in an increase in CO_2_ emissions and release of harmful chemicals into the environment (22). MICP can reduce this environmental burden by producing buildings that are more durable, prolonging the life of existing buildings, and improving soil properties without the use of toxic and hazardous chemical additives. Moreover, technologies that actively remove CO_2_ from the environment and sequestrate it in harmless or even useful minerals such as calcite will be critical in meeting zero-emission goals in the future.

## MATERIALS AND METHODS

### Bacterial isolates and growth.

Sample collection was from six locations across the United Kingdom, which included limestone caves and immature calcareous soils (Mendip Hills, England); soil, rock, and limestone caves (Bath, England); soil and rock scrapings (Monmouthshire, Wales); and soil and scrapings from marine rock in two locations in Cornwall (Mount’s Bay and Falmouth). Samples were stored at 4°C until use. Of each sample, 0.5 g was resuspended in 1 ml sterile saline solution (0.85% [wt/vol] NaCl) and heated to 80°C for 20 min to enrich for spore formers. Selection for alkali-tolerant bacteria was carried out by plating 100 μl of this suspension onto 0.25× B4 medium (1 g · liter^−1^ yeast extract, 12 g · liter^−1^ Trizma base, 1.25 g · liter^−1^ glucose, 2.5 g · liter^−1^ calcium acetate [Ca(OAc)_2_], and 15 g · liter^−1^ agar) adjusted with NaOH to pH 9. Glucose and Ca(OAc)_2_ were filter sterilized and added after autoclaving. Individual colonies with unique colony morphology were restreaked onto 0.25× B4 medium buffered with 75 mM *N*-cyclohexyl-2-aminoethanesulfonic acid (CHES) and 75 mM *N*-cyclohexyl-3-aminopropanesulfonic acid (CAPS), adjusted with NaOH to pH 10. Strains capable of growth at pH 10 and with visible crystals on the colony or in the surrounding agar were selected for further characterization. Single colonies were inoculated in lysogeny broth (LB; 10 g · liter^−1^ tryptone, 5 g · liter^−1^ yeast extract, and 10 g · liter^−1^ NaCl) and grown overnight before storage at −80°C in a 25% (wt/vol) glycerol solution. Isolates were subsequently maintained on LB agar. Sporosarcina pasteurii DSM33 was included as a reference organism and maintained on LB agar supplemented with 20 g · liter^−1^ urea (filter sterilized, added after autoclaving). All bacterial cultures were grown at 30°C, and liquid cultures were agitated at 150 rpm. Growth of liquid cultures was monitored spectrophotometrically by optical density at 600 nm (OD_600_) in cuvettes with a 1-cm light path length. Representative isolates showing the best characteristics were deposited into the Deutsche Sammlung von Mikroorganismen und Zellkulturen (DSMZ) collection (Germany) under the following accession numbers: DSM 110488 (RC1_1), DSM 110495 (PD1_1), DSM 110490 (EM1), DSM 110491 (CG7_3), DSM 110492 (CG7_2), DSM 110493 (CG6_1), DSM 110494 (CG4_2), and DSM 110489 (MM1_1).

### Identification of isolates and phylogenetic analysis.

The 16S rRNA gene fragment was amplified using the 27F (5′-AGAGTTTGATCMTGGCTCAG-3′) and 1492R (5′-TACCTTGTTACGACTT-3′) primers (26). Amplification was performed in a total volume of 25 μl containing 12.5 μl of 2× OneTaq Mastermix (New England Biolabs [NEB]), 9.9 μl of nuclease-free H_2_O, 2.4% (vol/vol) dimethyl sulfoxide (DMSO), 0.2 μM each primer, and 1 μl of an overnight culture as the template. Reaction conditions were as follows: initial denaturation at 94°C for 5 min followed by 30 cycles consisting of denaturation at 94°C for 30 s, annealing at 45°C for 30 s, and extension at 68°C for 2 min. The final extension was at 68°C for 5 min. For enzymatic cleanup, 2 μl of EXO-SAP (100 U shrimp alkaline phosphatase [Thermo Scientific], 100 U exonuclease I [Thermo Scientific], and 895 μl nuclease-free H_2_O) was added to every 5 μl of PCR product, and the reaction mixture was incubated at 37°C for 15 min before enzyme inactivation at 80°C for 15 min. Sequencing of PCR products was carried out using the chain termination method (Eurofins Genomics, Germany). In the case of strains where direct sequencing of the PCR product was unsuccessful, 16S rRNA gene fragments were first cloned into the Topo vector according to the manufacturer’s instructions (TOPO TA cloning kit, Invitrogen). Sequencing of the resulting plasmids was performed using the M13 forward primer (5′-CCCAGTCACGACGTTGTAAAACG-3′). BioEdit (27) was used to assemble sequences obtained from forward and reverse sequencing reactions. Sequences were compared against the nonredundant GenBank nucleotide collection using blastn (https://blast.ncbi.nlm.nih.gov/Blast.cgi). For phylogenetic analyses, the 16S rRNA gene sequences of nearest relatives for each strain according to blastn analysis, as well as that of Sporosarcina pasteurii DSM 33, were obtained from GenBank, and evolutionary analyses were carried out in MEGA 7.0 (28). Sequences were aligned using MUSCLE, and conserved blocks were selected from these multiple alignments using GBlocks (29). Phylogenetic trees were constructed using the maximum likelihood (ML) method based on the Tamura-Nei model (30). Bootstrap values were inferred from 1,000 replicates, and partitions reproduced in less than 50% of the bootstrap replicates were collapsed.

### Analysis of calcium carbonate precipitation.

Spatial distribution of calcite precipitation and pH changes was assessed by spotting 20 μl of an overnight culture (OD_600_ adjusted to 1) onto nutrient agar (23 g · liter^−1^; Oxoid,) supplemented with phenol red (0.025 g · liter^−1^) and Ca(OAc)_2_ (2.5 g · liter^−1^ or 10 g · liter^−1^), with or without urea (20 g · liter^−1^). Physical appearance of plates and crystals was recorded photographically at 1, 3, 5, 24, and 48 h.

The rate of mineral precipitation over time was determined by inoculating (1:1,000) 150 ml LB broth supplemented with 10 g · liter^−1^ Ca(OAc)_2_ from overnight cultures and monitoring viable cell numbers, pH, and insoluble calcium precipitated over time. Viable cells were determined as CFU by the plate count method. pH was recorded from aliquots of the culture using a pH electrode (Jenway 924 030; Cole-Parmer, Staffordshire UK) coupled to a pH meter (Jenway 3510 pH meter). Insoluble precipitate was recovered by centrifugation (975 × *g* for 2 min at room temperature [RT]) and washed three times in 50 ml distilled water to remove planktonic cells and culture medium before oven drying at 50°C for 48 h. Morphology of dried precipitates was examined at accelerating voltages ranging from 10 to 20 kV by scanning electron microscopy (JSM 6480LV; JEOL, Welwyn, UK) equipped with an energy-dispersive X-ray (EDX) analyzer for elemental analysis. Representative samples were prepared by spreading dried precipitate on double-sided carbon tape placed on aluminum stubs. For EDX, samples were placed under vacuum overnight before analysis. For imaging, samples were gold coated by sputtering for 3 min at RT. Samples imaged with field emission electron microscopy (FE-SEM) (JSM-6301F cold field emission SEM, JEOL) were prepared in a similar manner as those for SEM but coated in chromium to a thickness of 20 nm and examined at 5 kV.

### Urease activity.

Qualitative urease activity of bacterial isolates was determined using urease test broth (0.1 g · liter^−1^ yeast extract, 20 g · liter^−1^ urea, 0.01 g · liter^−1^ phenol red, 0.67 mM KH_2_PO_4_, and 0.67 mM Na_2_HPO_4_ [pH 6.8 ± 0.2]). Test broth (2 ml) was inoculated with 100 μl of an overnight culture grown in LB and incubated at 30°C for up to 5 days. A urease-positive reaction was characterized by a change in color from yellow/orange to pink.

For quantitative urease measurements in whole cells, cultures were grown at 30°C with agitation (150 rpm). Overnight cultures (20 μl, LB broth) were used to inoculate 25 ml (1:500) each of low-nitrogen (0.2 g · liter^−1^ yeast extract, 0.125 g · liter^−1^ peptone, and 2.5 g · liter^−1^ NaCl) and standard medium (2 g · liter^−1^ yeast extract, 1.25 g · liter^−1^ peptone, 2.5 g · liter^−1^ NaCl) broths. Where preinduction by urea was required, broths were supplemented with urea (20 g · liter^−1^). Cultures were grown (for 24 h) and cells were harvested from 1 ml of culture by centrifugation (8,000 × *g*, 2 min). Cells were resuspended in 1 volume of 0.1 M potassium phosphate buffer (pH 8.0), and the OD_600_ was recorded. Depending on the urease activities of individual strains, dilutions of cells were made to ensure the activity was within the linear range of the assay. These dilutions were recorded and accounted for during OD normalization. Urease activity was determined according to the phenol-hypochlorite method (31), adapted from Achal et al. (32) for whole cells. The reaction mix volumes were multiplied by the number of time points in the assay to allow one larger-volume mix to be prepared. Per time point, individual reaction mixes contained 75 μl cell suspension, 300 μl 0.1 M potassium phosphate buffer (pH 8.0), and 750 μl 0.1 M urea solution. The mixture was incubated at 30°C, and 1-ml aliquots were removed at 0, 30, 60, 90, and 120 min. At each time point, the reaction was stopped by adding 300 μl phenol nitroprusside and 300 μl alkaline hypochlorite. The final color was developed at 37°C for 25 min, and absorbance was measured at 625 nm. Ammonium chloride (25 to 2,500 μM) solutions were used to produce a standard curve (Fig. S1). One unit of activity (U) was defined as the release of 1 μmol ammonia per minute, which was normalized to the optical density and volume of cell suspension used (U · OD^−1^ · ml^−1^).

### Endospore production.

Endospores of bacterial strains were prepared by inoculating 150 ml LB broth (1:1,000) from an overnight culture grown at 30°C with shaking (150 rpm). This culture was again grown overnight, and then cells were harvested by centrifugation (3,200 × *g*, 10 min at RT) and resuspended in 750 ml Difco sporulation medium (DSM; 8 g · liter^−1^ nutrient broth [Oxoid], 13.41 mM KCl, and 0.49 mM MgSO_4_, adjusted to pH 7.6 with NaOH). Prior to use, 1 mM Ca(NO_3_)_2_, 0.01 mM MnCl_2_, and 1 μM FeSO_4_ were added from a filter-sterilized stock solution (33). Cultures were grown with agitation (150 rpm) at 30°C for at least 48 h before sporulation was assessed using phase-contrast microscopy. When the majority of cells contained phase-bright endospores, cells were pelleted by centrifugation (3,200 × *g*, 10 min at RT) and washed thrice in 10 mM Tris-HCl (pH 9), followed by 30 min of treatment with chlorhexidine digluconate (0.3 mg · ml^−1^) to kill vegetative cells. Washing was repeated as before, and spore pellets were snap-frozen in liquid nitrogen and freeze-dried under vacuum overnight.

### Preparation of mortar samples.

Mortar prisms (65 mm by 40 mm by 40 mm) were comprised of two layers. The first contained (per 3 prisms): 253.3 g sand conforming to standard BS EN 196-1, 92 g Portland limestone cement (CEM II/A-L 32.5R), 46 g water, 1 g yeast extract, 4.55 g calcium nitrate, and 3.54 g aerated concrete granules. Mortars containing ureolytic bacteria also contained urea (3.68 g per 3 prisms). Spores (2.1 × 10^10^ CFU) were resuspended in 1 ml distilled water (dH_2_O) and imbibed into the aerated concrete granules before drying and sealing with polyvinyl acetate (PVA; 30% [wt/wt]). After approximately 3 h, the second, top layer was cast. The second layer contained standard cement mortar (per 3 prisms: 276 g standard sand, 92 g cement, and 46 g water). Reference specimens were cast in two layers but contained only standard cement mortar. Specimens remained at room temperature for 24 to 48 h before demolding and subsequent curing for 28 days submersed in tap water. After curing, specimens were oven dried at 50°C for 24 h. The top third of the prism was wrapped with carbon fiber-reinforced polymer strips to enable generation of a crack of controlled width. A notch (1.5 mm deep) was sawed at midspan to serve as a crack initiation point. Specimens were cracked by 3-point bending using a 30-kN Instron static testing frame. A crack mouth opening displacement (CMOD) gauge was used to measure crack width. Load was applied to maintain a crack growth of 25 μm per minute, and loading was stopped when the crack width was predicted to be 500 μm wide after load removal. A marker pen was used to indicate specific crack sections to enable monitoring of the crack at the same site. Following cracking, prisms were placed in tanks that were open to the atmosphere and filled with tap water to 1 cm below the top of the mortars and then incubated at room temperature for 2 months. Visualization of crack healing was monitored using a Leica M205C light microscope, and images were taken of freshly cracked mortars and after 1, 4, and 8 weeks of healing.

### Water permeability tests.

Water flow rate before cracking, after cracking, and after 8 weeks of healing was determined to monitor effects of cracking and healing on water permeability in mortars, as described previously (34). The instrument used was based on RILEM test method II.4 (35). The bottom of a 10-ml measuring cylinder (0.2-ml graduation) was removed, and a 40-mm polyvinyl chloride (PVC) pipe with a 15-mm depth was fixed to the bottom. This was sealed onto the mortar with glue. This cylinder was filled with water, and the time it took to drop from initial height *h*_1_ to final height *h*_2_ was recorded. The total height was either 78.5 mm or, in the case of samples with low permeability, the height to which the water level had dropped after 30 min. The permeability coefficient was calculated according to equation 3 (34).

(3) $$k=\frac{\mathrm{aL}}{\mathrm{At}}\text{ln}\left[\frac{h_{1}}{h_{2}}\right]$$

where *k* = water permeability coefficient (cm/s); *a* = cross-sectional area of the cylinder (1.77 cm^2^); *L* = thickness of specimen (4 cm); *A* = cross-sectional area of the specimen which equals the cross-sectional area of acrylic plate (10.18 cm^2^); *t* = time (s); *h*_1_ = initial water head (12.4 cm); and *h*_2_ = final water head (cm). The percentage of crack healing was calculated according to equation 4:

(4) $$\text{Healing percentage}\;\left(\%\right)=\frac{\left(k_{0}-k_{t}\right)}{k_{0}}\;\times \;100$$

where *k*_0_ = initial water permeability after cracking and *k_t_* = water permeability at healing time *t*.

## Supplementary Material

Supplemental file 1

## References

[1] IPCC. 2018 Summary for policymakers. *In* Masson-Delmotte V, Zhai P, Pörtner HO, Roberts D, Skea J, Shukla PR, Pirani A, Moufouma-Okia W, Péan C, Pidcock R, Connors S, Matthews JBR, Chen Y, Zhou X, Gomis MI, Lonnoy E, Maycock T, Tignor M, Waterfield T (ed), Global warming of 1.5°C An IPCC special report on the impacts of global warming of 1.5°C above pre-industrial levels and related global greenhouse gas emission pathways, in the context of strengthening the global response to the threat of climate change, sustainable development, and efforts to eradicate poverty. World Meteorological Organization, Geneva, Switzerland https://www.ipcc.ch/sr15/.

[2] Mitchell AC, Dideriksen K, Spangler LH, Cunningham AB, Gerlach R 2010 Microbially enhanced carbon capture and storage by mineral-trapping and solubility-trapping. Environ Sci Technol 44:5270–5276. doi:10.1021/es903270w. 20540571

[3] Altermann W, Kazmierczak J, Oren A, Wright DT 2006 Cyanobacterial calcification and its rock-building potential during 3.5 billion years of Earth history. Geobiology 4:147–166. doi:10.1111/j.1472-4669.2006.00076.x.

[4] Wilmeth DT, Johnson HA, Stamps BW, Berelson WM, Stevenson BS, Nunn HS, Grim SL, Dillon ML, Paradis O, Corsetti FA, Spear JR 2018 Environmental and biological influences on carbonate precipitation within hot spring microbial mats in Little Hot Creek, CA. Front Microbiol 9:1464. doi:10.3389/fmicb.2018.01464. 30057571 PMC6053513

[5] Achal V, Pan X, Zhang D 2011 Remediation of copper-contaminated soil by *Kocuria flava* CR1, based on microbially induced calcite precipitation. Ecol Eng 37:1601–1605. doi:10.1016/j.ecoleng.2011.06.008.

[6] Ceci A, Pinzari F, Riccardi C, Maggi O, Pierro L, Papini MP, Gadd GM, Persiani AM 2018 Metabolic synergies in the biotransformation of organic and metallic toxic compounds by a saprotrophic soil fungus. Appl Microbiol Biotechnol 102:1019–1033. doi:10.1007/s00253-017-8614-9. 29138910

[7] Souto-Martinez A, Arehart JH, Srubar WV III 2018 Cradle-to-gate CO_2_e emissions vs. *in situ* CO_2_ sequestration of structural concrete elements. Energy Build 167:301–311. doi:10.1016/j.enbuild.2018.02.042.

[8] Huntzinger DN, Eatmon TD 2009 A life-cycle assessment of Portland cement manufacturing: comparing the traditional process with alternative technologies. J Clean Prod 17:668–675. doi:10.1016/j.jclepro.2008.04.007.

[9] Boquet E, Boronat A, Ramos-Cormenzana A 1973 Production of calcite (calcium carbonate) crystals by soil bacteria is a general phenomenon. Nature 246:527–529. doi:10.1038/246527a0.

[10] Hammes F, Verstraete W 2002 Key roles of pH and calcium metabolism in microbial carbonate precipitation. Rev Environ Sci Biotechnol 1:3–7. doi:10.1023/A:1015135629155.

[11] Power IM, Wilson SA, Small DP, Dipple GM, Wan W, Southam G 2011 Microbially mediated mineral carbonation: roles of phototrophy and heterotrophy. Environ Sci Technol 45:9061–9068. doi:10.1021/es201648g. 21879741

[12] Zhu T, Dittrich M, Dittrich M 2016 Carbonate precipitation through microbial activities in natural environment, and their potential in biotechnology: a review. Front Bioeng Biotechnol 4:4. doi:10.3389/fbioe.2016.00004. 26835451 PMC4718973

[13] Zhao Q, Li L, Asce M, Li C, Li M, Amini F, Asce F, Zhang H 2014 Factors affecting improvement of engineering properties of MICP-treated soil catalyzed by bacteria and urease. J Mater Civ Eng 26:1–10.

[14] Cheng L, Cord-Ruwisch R 2014 Upscaling effects of soil improvement by microbially induced calcite precipitation by surface percolation. Geomicrobiol J 31:396–406. doi:10.1080/01490451.2013.836579.

[15] Bang SS, Lippert JJ, Yerra U, Mulukutla S, Ramakrishnan V 2010 Microbial calcite, a bio-based smart nanomaterial in concrete remediation. Int J Smart Nano Mater 1:28–39. doi:10.1080/19475411003593451.

[16] Wang J, De Belie N, Verstraete W 2012 Diatomaceous earth as a protective vehicle for bacteria applied for self-healing concrete. J Ind Microbiol Biotechnol 39:567–577. doi:10.1007/s10295-011-1037-1. 21927907

[17] Wang J, Jonkers HM, Boon N, De Belie N 2017 *Bacillus sphaericus* LMG 22257 is physiologically suitable for self-healing concrete. Appl Microbiol Biotechnol 101:5101–5114. doi:10.1007/s00253-017-8260-2. 28365797

[18] Jonkers HM, Thijssen A, Muyzer G, Copuroglu O, Schlangen E 2010 Application of bacteria as self-healing agent for the development of sustainable concrete. Ecol Eng 36:230–235. doi:10.1016/j.ecoleng.2008.12.036.

[19] Sharma TK, Alazhari M, Heath A, Paine K, Cooper RM 2017 Alkaliphilic *Bacillus* species show potential application in concrete crack repair by virtue of rapid spore production and germination then extracellular calcite formation. J Appl Microbiol 122:1233–1244. doi:10.1111/jam.13421. 28199767

[20] Alazhari M, Sharma T, Heath A, Cooper R, Paine K 2018 Application of expanded perlite encapsulated bacteria and growth media for self-healing concrete. Constr Build Mater 160:610–619. doi:10.1016/j.conbuildmat.2017.11.086.

[21] Mobley HLT, Hausinger RP 1989 Microbial ureases: significance, regulation, and molecular characterization. Microbiol Rev 53:85–108. doi:10.1128/MMBR.53.1.85-108.1989. 2651866 PMC372718

[22] DeJong JT, Mortensen BM, Martinez BC, Nelson DC 2010 Bio-mediated soil improvement. Ecol Eng 36:197–210. doi:10.1016/j.ecoleng.2008.12.029.

[23] Schultze-Lam S, Fortin D, Davis BS, Beveridge TJ 1996 Mineralization of bacterial surfaces. Chem Geol 132:171–181. doi:10.1016/S0009-2541(96)00053-8.

[24] Stocks-Fischer S, Galinat JK, Bang SS 1999 Microbiological precipitation of CaCO_3_. Soil Biol Biochem 31:1563–1571. doi:10.1016/S0038-0717(99)00082-6.

[25] Tobler DJ, Cuthbert MO, Greswell RB, Riley MS, Renshaw JC, Handley-Sidhu S, Phoenix VR 2011 Comparison of rates of ureolysis between *Sporosarcina pasteurii* and an indigenous groundwater community under conditions required to precipitate large volumes of calcite. Geochim Cosmochim Acta 75:3290–3301. doi:10.1016/j.gca.2011.03.023.

[26] Lane D 1991 16S/23S rRNA sequencing, p 115–175. *In* Stackebrandt E, Goodfellow M (ed), Nucleic acid techniques in bacterial systematics. John Wiley and Sons, Chichester, UK.

[27] Hall T 1999 BioEdit: a user-friendly biological sequence alignment editor and analysis program for Windows 95/98/NT. Nucleic Acids Symp Ser (Oxf) 41:95–98.

[28] Kumar S, Stecher G, Tamura K 2016 MEGA7: Molecular Evolutionary Genetics Analysis version 7.0 for bigger datasets. Mol Biol Evol 33:1870–1874. doi:10.1093/molbev/msw054. 27004904 PMC8210823

[29] Castresana J 2000 Selection of conserved blocks from multiple alignments for their use in phylogenetic analysis. Mol Biol Evol 17:540–552. doi:10.1093/oxfordjournals.molbev.a026334. 10742046

[30] Tamura K, Nei M 1993 Estimation of the number of nucleotide substitutions in the control region of mitochondrial DNA in humans and chimpanzees. Mol Biol Evol 10:512–526. doi:10.1093/oxfordjournals.molbev.a040023. 8336541

[31] Natarajan KR 1995 Kinetic study of the enzyme urease from *Dolichos biflorus*. J Chem Educ 72:556–557. doi:10.1021/ed072p556.

[32] Achal V, Mukherjee A, Basu PC, Reddy MS 2009 Lactose mother liquor as an alternative nutrient source for microbial concrete production by *Sporosarcina pasteurii*. J Ind Microbiol Biotechnol 36:433–438. doi:10.1007/s10295-008-0514-7. 19107535

[33] Sonenshein AL, Cami B, Brevet J, Cote R 1974 Isolation and characterization of rifampin-resistant and streptolydigin-resistant mutants of *Bacillus subtilis* with altered sporulation properties. J Bacteriol 120:253–265. doi:10.1128/JB.120.1.253-265.1974. 4370705 PMC245758

[34] Lee YS, Ryou JS 2016 Crack healing performance of PVA-coated granules made of cement, CSA, and Na_2_CO_3_ in the cement matrix. Materials 9:555–575. doi:10.3390/ma9070555. PMC5456870 28773677

[35] RILEM. 1987 Measurement of water absorption under low pressure. RILEM test method no. 11.4.

