# Supplementary material for: In-Depth Profiling of Calcite Precipitation by Environmental Bacteria Reveals Fundamental Mechanistic Differences with Relevance to Application
Source: Appl Environ Microbiol. 2020 Mar 18;86(7):e02739-19. doi: 10.1128/AEM.02739-19 (PMC7082560; doi:10.1128/AEM.02739-19)
Supplement: Supplemental file 1 [file AEM.02739-19-s0001.pdf]

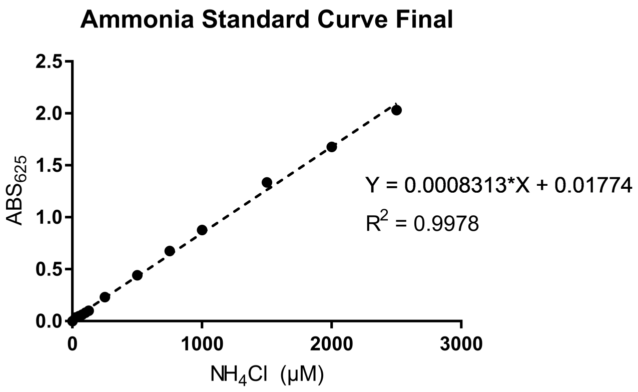

Figure S1. Ammonium chloride concentration vs absorbance standard curve. Ammonium chloride (25-2500 µM) solutions were used as standards to quantify the change in absorbance at 625 nm wavelength (A_{625}) observed using phenol-hypochlorite as described in methods. The linear fit of ammonium chloride concentration versus absorbance used to calculate unknown concentrations is shown by the equation and dashed line.

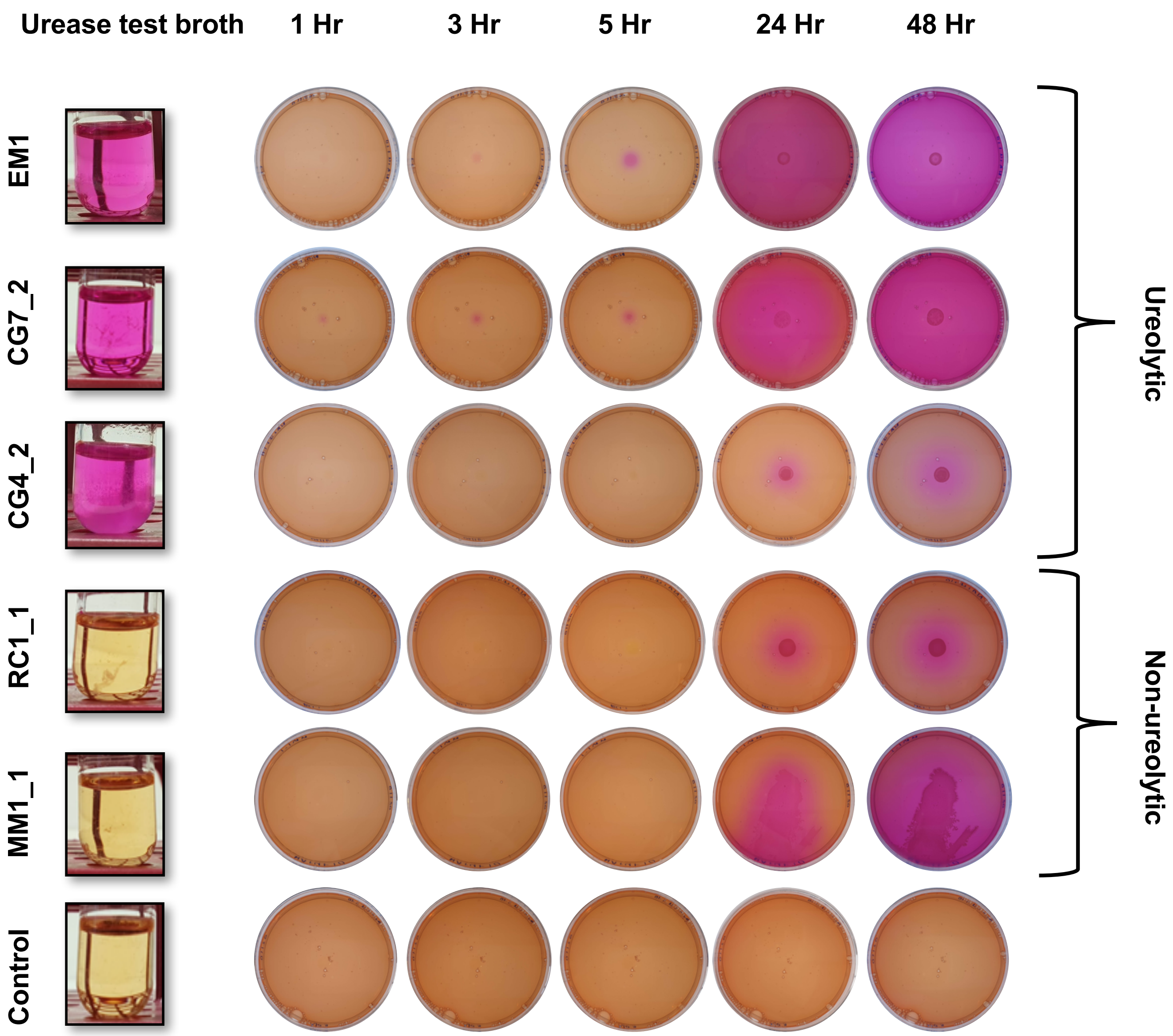

Figure S2. Diversity in ureolytic activity across environmental isolates. *Left*, urease test broth inoculated with the strains indicated to the left. *Right*, nutrient agar plates supplemented with phenol red, calcium acetate, and urea were inoculated in the centre with suspensions of the same strains. Pink shading indicates the pH change caused by ammonia release from ureolytic activity. Please note that MM1_1 is non-ureolytic. The more widely distributed pink colour change observed for this isolate is due to a swarming behavior, and hence more widely distributed bacterial growth, it displayed on the urea containing media.

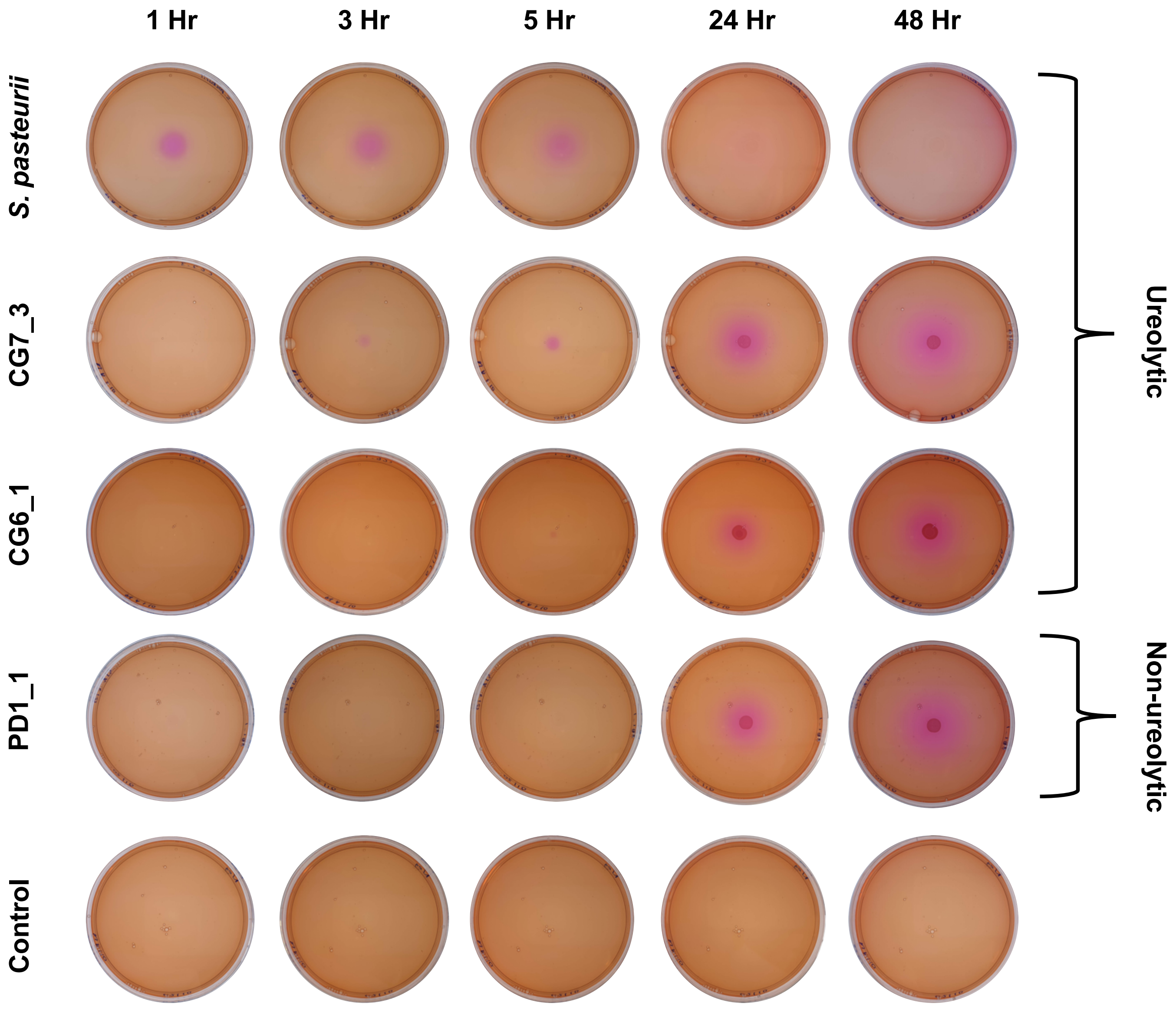

Figure S3. No pH changes are seen in ureolytic isolates when urea is excluded from the media. Nutrient agar plates supplemented with phenol red and calcium acetate were inoculated in the centre with suspensions of the same strains. Only local pH changes characteristic of normal metabolism are seen.

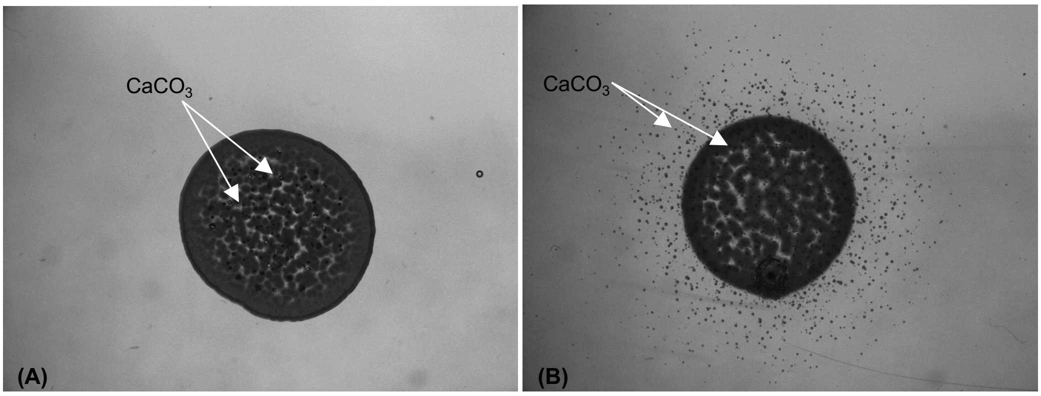

Figure S4. Comparison of crystal localisation in ureolytic strain CG7_3 without (A) and with (B) urea. Ureolytic bacteria precipitate crystals locally in the absence of urea, similar to what is seen in non-ureolytic strains. Arrows indicate $CaCO_3$ crystals.

Fig S4

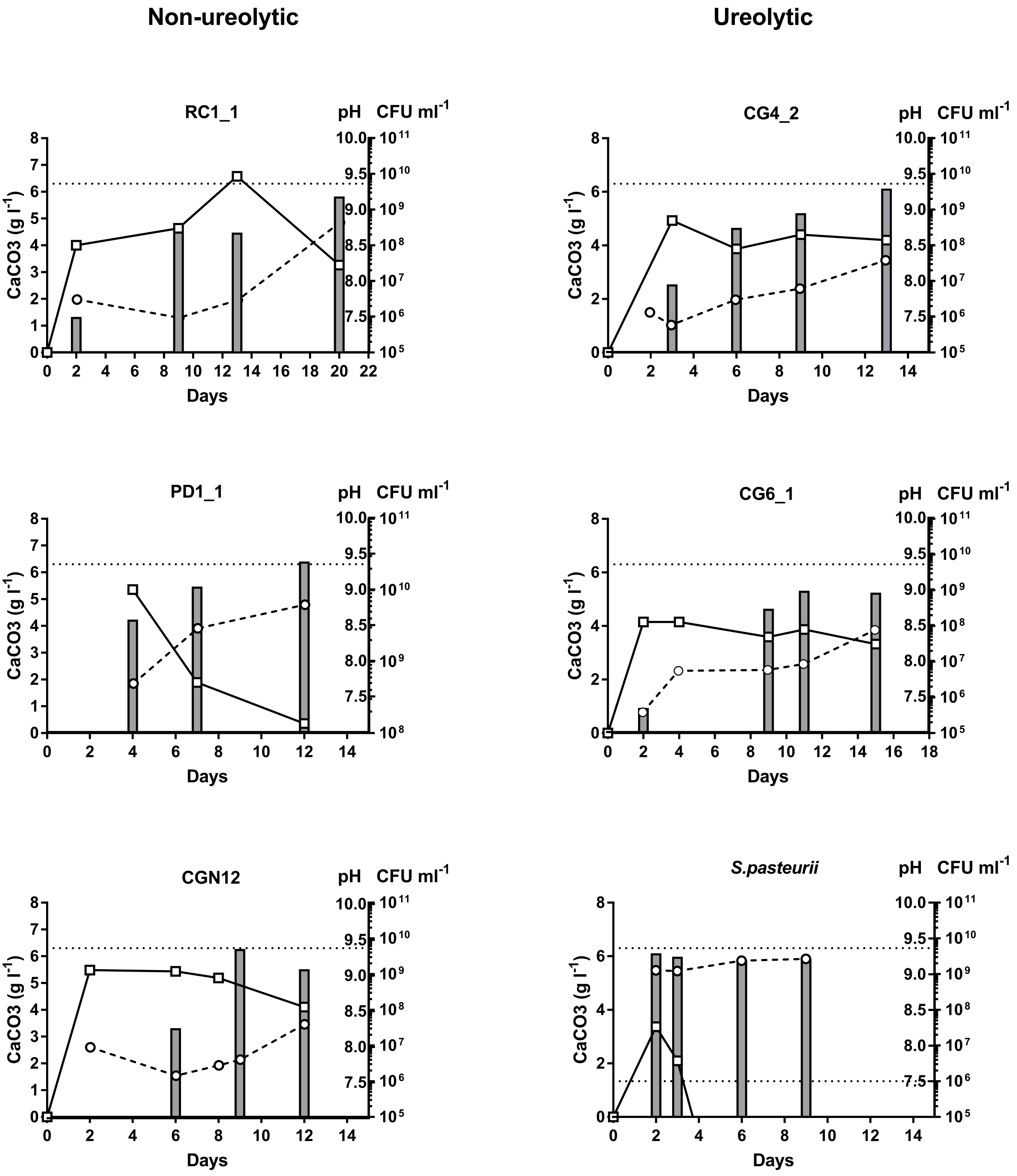

Figure S6. EDX analyses of precipitates produced by environmental bacteria. *Left,* the position on the precipitate where EDX was conducted (purple boxes). *Centre*, corresponding EDX spectra are shown, with the three peaks of main components labelled below. *Right,* elemental analysis indicating that the three main components (%) of the precipitate where carbon (C), oxygen (O), and calcium (Ca). Minor elements were excluded from the summary tables.

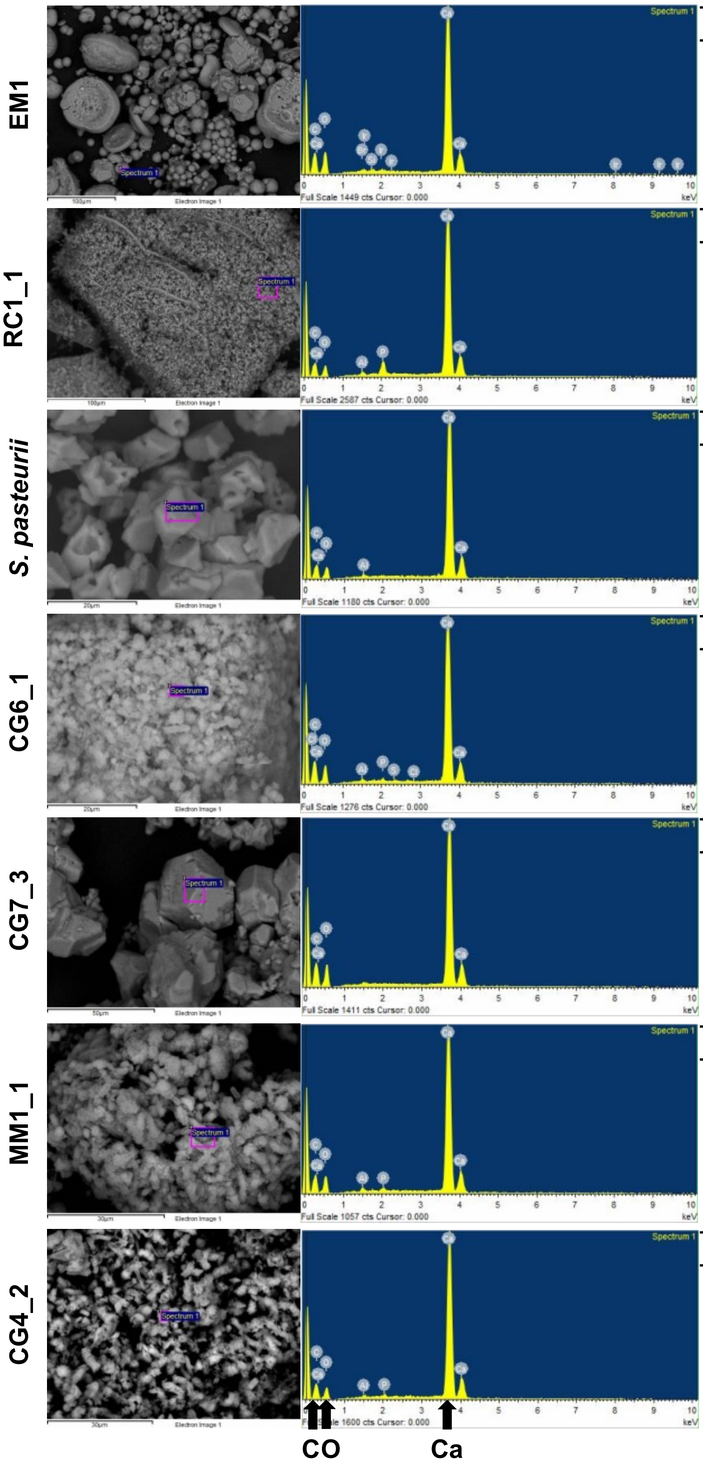

EM1

Element	Weight%	Atomic%
C	18.32	30.10
O	40.64	50.12
Ca	39.33	19.36
**Totals**	**98.29**	**99.58**

RC1_1

Element	Weight%	Atomic%
C	17.80	31.62
O	30.01	40.02
Ca	48.85	26.00
**Totals**	**96.66**	**97.64**

S. pasteurii

Element	Weight%	Atomic%
C	15.27	27.27
O	33.90	45.44
Ca	50.50	27.03
**Totals**	**99.67**	**99.74**

CG6_1

Element	Weight%	Atomic%
C	20.26	33.38
O	36.40	45.03
Ca	42.26	20.87
**Totals**	**98.92**	**99.28**

CG7_3

Element	Weight%	Atomic%
C	20.38	32.83
O	39.55	47.83
Ca	40.08	19.35
**Totals**	**100.00**	**100.00**

MM1_1

Element	Weight%	Atomic%
C	18.06	30.50
O	36.47	46.25
Ca	44.29	22.42
**Totals**	**98.82**	**99.17**

CG4_2

Element	Weight%	Atomic%
C	17.69	31.06
O	32.09	42.31
Ca	49.25	25.92
**Totals**	**99.03**	**99.29**

Figure S6. EDX analyses of precipitates produced by environmental bacteria. *Left,* the position on the precipitate where EDX was conducted (purple boxes). *Centre*, corresponding EDX spectra are shown, with the three peaks of main components labelled below. *Right,* elemental analysis indicating that the three main components (%) of the precipitate where carbon (C), oxygen (O), and calcium (Ca). Minor elements were excluded from the summary tables.

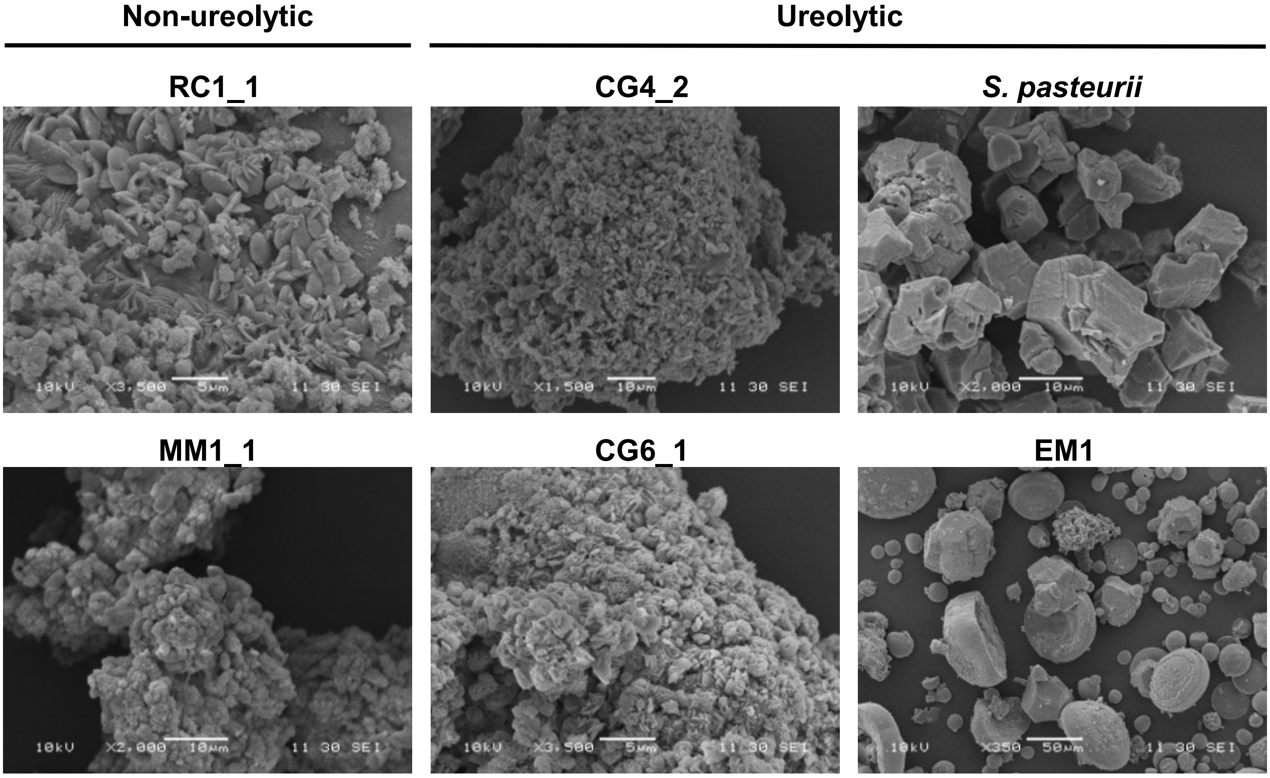

Figure S7. Crystal morphology in ureolytic and non-ureolytic isolates. Isolates were grown in LB medium supplemented with urea (20 g l^{-1}) and $Ca(OAc)_2$ (10 g l^{-1}) and electron micrographs of representative precipitates were taken between days 9-15. Strain names and their ureolytic potential are stated above.

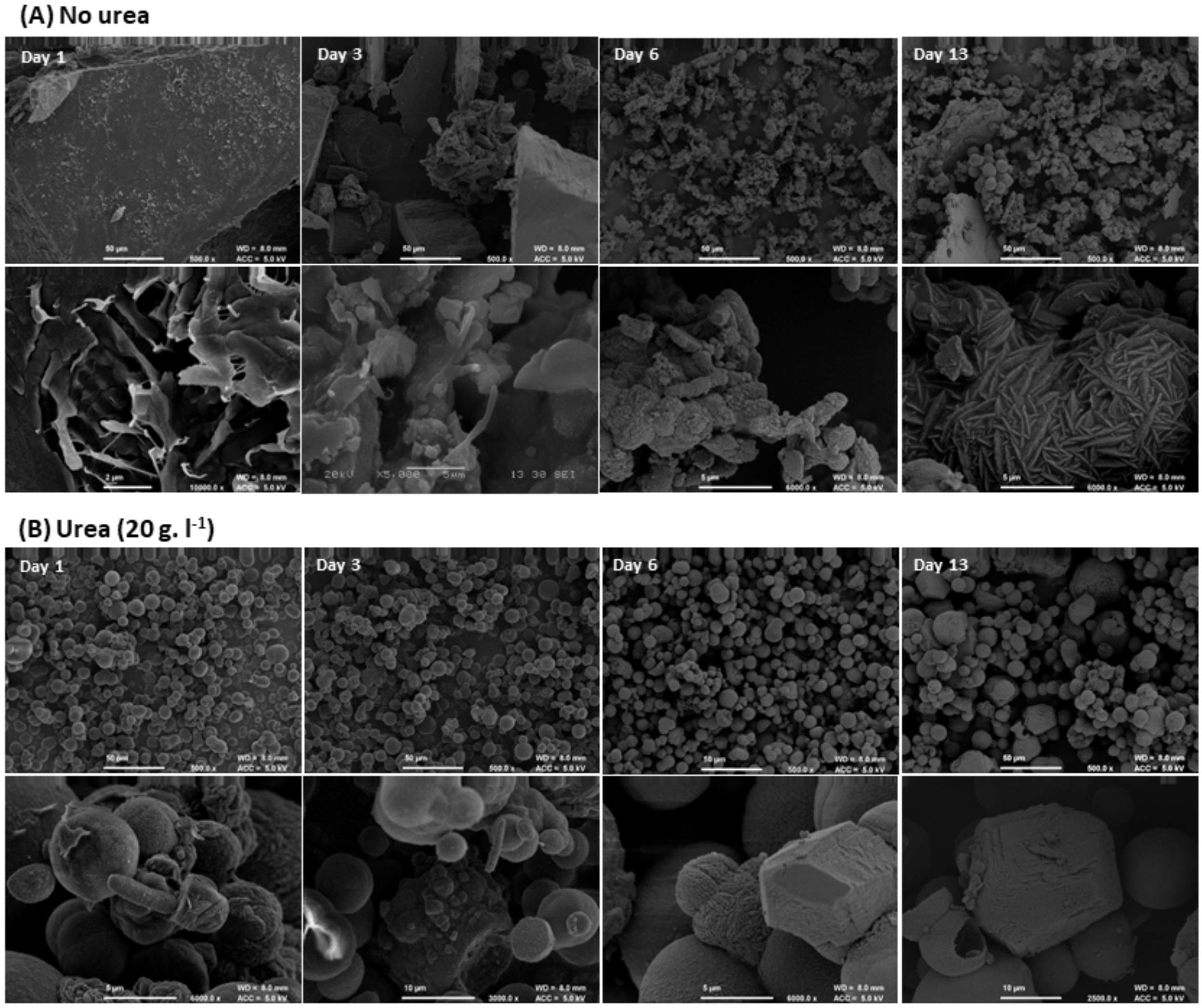

Figure S8. Comparison of crystal morphology in CG7_3 grown in the absence and presence of urea. The ureolytic strain CG7_3 was grown in LB medium supplemented with $Ca(OAc)_2$ (10 g l^{-1}) in the absence (A) and presence (B) of urea (20 g l^{-1}). Electron micrographs of precipitate were taken at days 1,3, 6 and 13. The top panel of each series represents a low magnification view, the bottom panel a high magnification close-up.

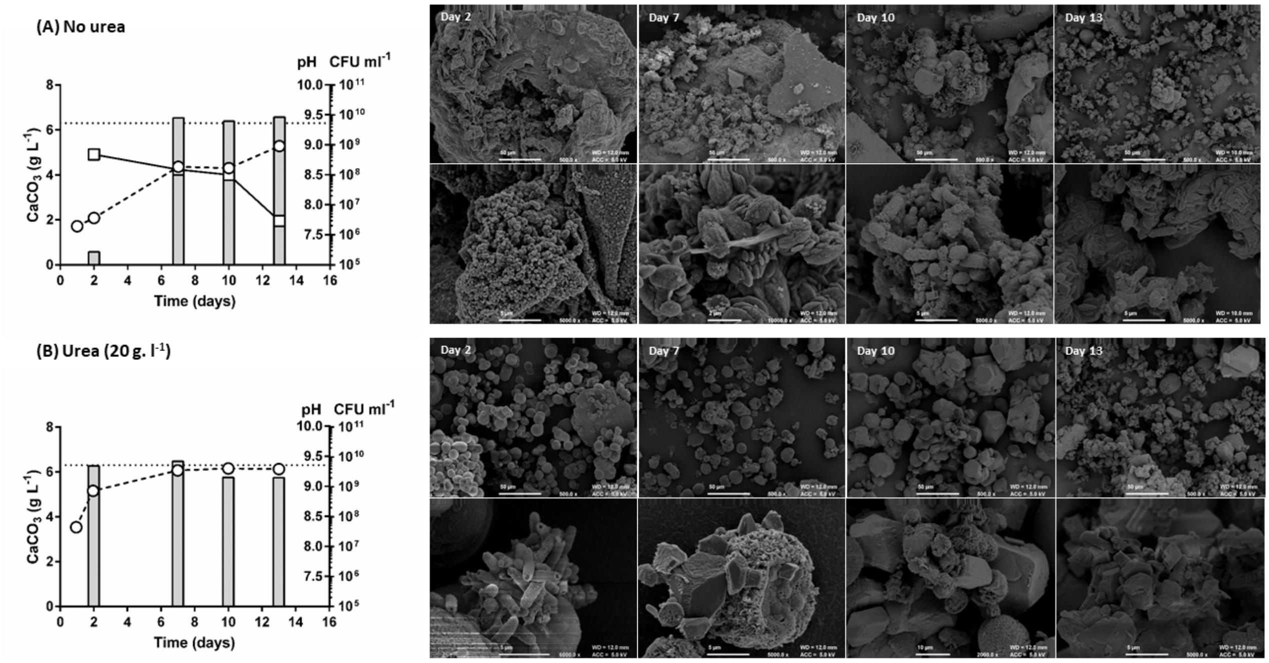

Figure S9. Comparison of ureolytic and non-ureolytic mechanisms of calcite precipitation in CG7_3. Ureolytic strain CG7_3 was grown in LB medium supplemented with $Ca(OAc)_2$ (10 g l^{-1}) in the absence (A) and presence (B) of urea (20 g l^{-1}). Precipitation of insoluble calcium carbonate (bars, g l^{-1}), pH changes (circles) and changes in cell number (boxes, CFU ml^{-1}) were monitored over time (days). *Right,* electron micrographs of representative precipitate taken at days 2, 7, 10 and 13.
